# A Single-Sensor Approach for Noninvasively Tracking Phase Velocity in Tendons during Dynamic Movement

**DOI:** 10.3390/mi15010032

**Published:** 2023-12-23

**Authors:** Dylan G. Schmitz, Darryl G. Thelen, Stephanie G. Cone

**Affiliations:** 1 Department of Mechanical Engineering, University of Wisconsin–Madison, Madison, WI 53706, USA; 2 Department of Biomedical Engineering, University of Wisconsin–Madison, Madison, WI 53706, USA; 3 Department of Biomedical Engineering, University of Delaware, Newark, DE 19713, USA; sgcone@udel.edu

**Keywords:** tendon mechanics, wave propagation, dispersion, shear wave tensiometry

## Abstract

Shear wave tensiometry is a noninvasive method for directly measuring wave speed as a proxy for force in tendons during dynamic activities. Traditionally, tensiometry has used broadband excitation pulses and measured the wave travel time between two sensors. In this work, we demonstrate a new method for tracking phase velocity using shaped excitations and measurements from a single sensor. We observed modulation of phase velocity in the Achilles tendon that was generally consistent with wave speed measures obtained via broadband excitation. We also noted a frequency dependence of phase velocity, which is expected for dispersive soft tissues. The implementation of this method could enhance the use of noninvasive wave speed measures to characterize tendon forces. Further, the approach allows for the design of smaller shear wave tensiometers usable for a broader range of tendons and applications.

## 1. Introduction

Characterizing the forces generated by the muscles and understanding how they are transmitted through the tendons during human movement is important for comprehending coordination patterns [1]. This knowledge has a wide range of practical applications, including evaluating an athlete’s performance and potential [2], predicting and identifying injuries [3], and guiding and monitoring rehabilitation [4]. One way to quantify these soft tissue forces is through the use of shear wave measurements. The basic idea is that by observing the speed of a wave as it travels through a structure, the axial stress in the soft tissue can then be inferred. Shear waves can be generated using a surface transducer and tracked with accelerometers attached to the skin. This technique, known as shear wave tensiometry, involves generating a wave in the tendon with a broadband pulse excitation. The speed of the wave is then calculated by measuring the time it takes to travel between two accelerometers with a known distance between them [5]. Unlike shear wave elastography, which is an ultrasonic technique used to measure soft tissue behavior [6,7,8], the shear wave tensiometry approach has a rapid update rate, making it suitable for tracking changes in tendon loading during dynamic movements with rapidly changing forces. Previous studies have successfully employed tensiometry to monitor tendon loading during various activities [9,10,11,12].

Two significant challenges are associated with the utilization of impulsive, broadband excitation in tensiometry. Firstly, it is important to note that, when it comes to wave propagation in biological soft tissue, a phenomenon known as dispersion occurs [13,14,15], meaning that the behavior of waves varies with different frequencies. The impulsive excitation often used in tensiometry triggers the inherent dynamics of the tissue structure, and this dynamic nature can change due to the load dependencies of the system impedance. As a result, the primary wave frequencies may fluctuate during dynamic loading [16], leading to variations in the measured wave speeds. One potential solution to this issue is to employ shaped excitations, which generate waves with a specific phase velocity at a well-defined frequency [17]. Secondly, when performing tensiometry measurements, it is essential that both the actuation and sensing components come into contact with the free tendon. Current tensiometer designs have a relatively large footprint, spanning approximately 30 mm from excitation input to final measurement output. While this size is suitable for longer structures like the Achilles tendon, it presents challenges when dealing with shorter structures that would benefit from a more compact sensor footprint, such as the patellar tendon in the knee or the ulnar collateral ligament in the elbow.

In this study, we present a novel approach to shear wave tensiometry that involves the utilization of shaped excitation pulses and a single-sensor technique for real-time tracking of in vivo tendon phase velocity. Our findings indicate that the application of shaped waveforms induces phase velocities with frequency dependence, in concordance with the inherent dispersion characteristics of the system. These outcomes have the potential to simplify and extend the application of shear wave tensiometry.

## 2. Methods

### 2.1. Wave Excitation and Measurement

**Figure 1:**
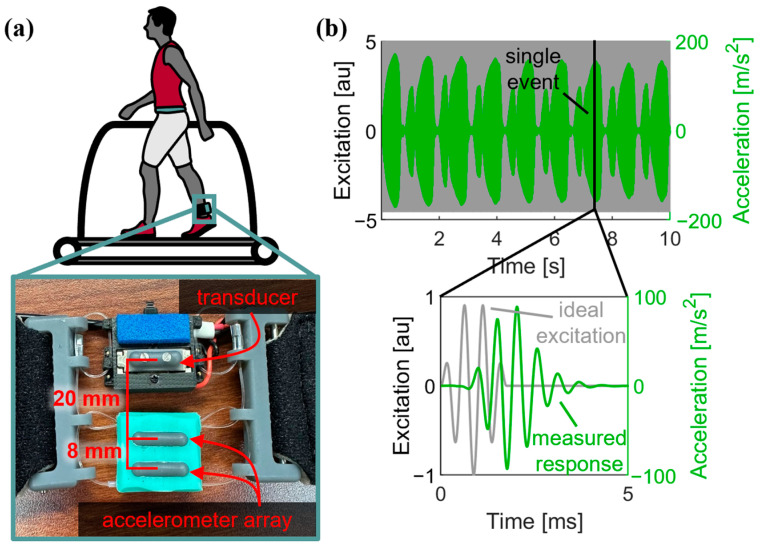
(**a**) Shear wave tensiometry was performed using a standard two-sensor device. The device was placed over the Achilles tendon. The transducer excited transverse waves and the propagation was measured with two accelerometers embedded in a silicone array. (**b**) Example of the first accelerometer measurements across a 10 s collection, with excitation wavelets introduced at 100 Hz. A single event consists of a modulated harmonic wavelet excitation and measured response.

Shear waves were excited in the Achilles tendon using a miniature surface transducer (Figure 1a). A shaped, discrete wavelet was chosen as the excitation signal such that the dominant harmonic component and amplitude modulation could be easily dissociated using a Hilbert transform. This input excitation ψ was defined by the following piecewise representation:

(1) $$\psi =\left\{\begin{array}{cc} A\cdot \sin\left(2\pi f_{A}t\right)\cdot \sin\left(2\pi f_{H}t+\varphi \right) & \;for\;0\leq t\leq t_{0}\\ 0 & otherwise \end{array}\right.$$

where A was the peak of the amplitude modulation and $f_{H}$ was the dominant wavelet frequency. The amplitude modulation was defined as a half-sine with the frequency (Equation (2)) and duration (Equation (3)) defined to produce a wavelet with seven lobes (Figure 1b).

(2) $$f_{A}=f_{H}/7$$

(3) $$t_{0}=3.5/f_{H}$$

Miniature skin-mounted accelerometers (PCB Piezotronics, Depew, NY, USA) were used to measure the transverse motion of the tendon at 20 mm and 28 mm from the transducer contact (Figure 1a).

### 2.2. Wave Speed Computation

**Figure 2:**
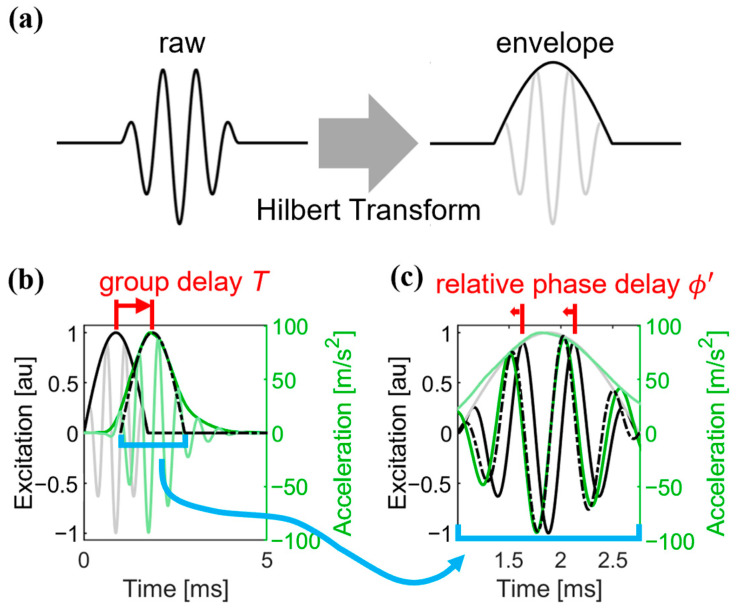
(**a**) The Hilbert transform extracted the envelope of the amplitude-modulated wavelets used to excite transverse waves in the tendon. (**b**) The group delay *T* optimally aligned the amplitudes of the envelope of the input wavelet and measured response. (**c**) The relative phase delay $\phi^{\prime }$ was then determined by correlating the measured response with phase-shifted reconstructions (range −π to π) of the time-shifted input to find the phase that maximized the correlation. The overall phase delay was the sum of the group delay (converted to radians) and the relative phase delay.

**Figure 3:**
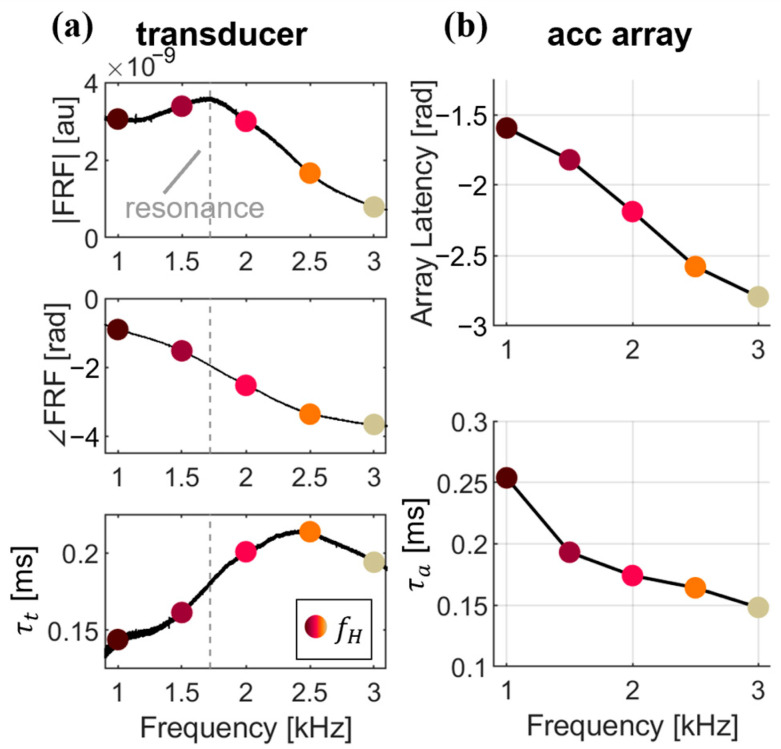
(**a**) System characterization of input transducer and resultant latencies. The magnitude ($\left|FRF\right|$) and phase (∠FRF) are resultant from standard frequency response function characterization. From ∠FRF, the transducer latency $\tau_{t}$ can be computed. (**b**) Quantification of the accelerometer array dynamics and associated latencies, represented as both phase angle in radians, and latency in milliseconds.

Wave speed was computed in two ways. The single-sensor method was applied only to the signal measured by the first accelerometer, and the timing of the wave arrival was quantified via both the group delay and relative phase shift. A two-step approach was implemented which distinguished the group and phase delays because in dispersive materials, these are known to be different [18]. To do this, the envelope of the measured acceleration was computed via a Hilbert transform [19] (Figure 2a). We then found the group delay T that maximized the normalized cross-correlation between the envelopes of the input excitation and the measured response (Figure 2b). The input excitation was then time-shifted by T. The relative phase shift was then determined by identifying that phase which maximized the correlation between the input excitation and measured response (Figure 2c). The overall phase delay ϕ was computed as the resultant sum of T represented as phase, and the relative phase delay [18]. Independent Kalman filters were implemented for both the group and phase delay computations. The a priori estimates of the system states were computed from the change in group and phase delay of consecutive events [20]. Phase velocity for the shear wave was computed from the known distance from the transducer to the accelerometer (*D* = 20 mm) and the arrival times (Equation (4)). The transducer latency ($\tau_{t}$) and the array latency ($\tau_{a}$) were subtracted from the overall phase delay (Figure 3).

(4) $$v_{p}=D/\left(T+\phi^{\prime }/2\pi f_{H}-\tau_{t}-\tau_{a}\right)$$

Dual-sensor wave speeds were computed as described previously [20]. Cross-correlations determined the between-sensor time delay and the between-event changes in time delay using signals captured by both accelerometers. A Kalman filter fused these measures to provide adjusted arrival times of the wave at each sensor. Wave speed was computed based on the known distance between accelerometers (8 mm) and the difference in wave arrival times at the two accelerometers.

### 2.3. System Latency Characterization

The frequency-based latencies for the transducer and the measurement array were determined independently. For the transducer, a system response characterization was performed by driving the transducer with a 0.1–5 kHz chirp with a duration of 60 s, and the resultant motion was measured with a miniature accelerometer affixed to the end effector (Figure 3a). To replicate the in vivo system behavior as closely as possible, the characterization was performed while the device was placed over an Achilles tendon during quiet standing. The built-in Matlab (Version R2023b) function “modalfrf” was used to compute the amplitude and phase response of the system. The frequency-dependent latency $\tau_{t}$ of the transducer in the time domain was computed from the phase response (∠FRF) and the frequency ($f_{H}$) (Equation (5)).

(5) $$\tau_{t}=\left(∠FRF\right)/f_{H}$$

The array latencies $\tau_{a}$ were determined by exciting an unloaded silicone block with five discrete wavelet frequencies, from 1–3 kHz in 0.5 kHz increments. A laser Doppler vibrometer (PDV-100, Polytec, Baden-Württemberg, Germany) targeted a fixed location relative to excitation on the silicone block and measured the transmitted wavelet motion. The test was repeated with the accelerometer array placed at the same distance from the excitation, and the vibrometer measured the velocity of the array. The difference in wave arrival time with and without the array was determined to be the additional delay introduced to the tensiometer measurement by the array system dynamics for each frequency (Figure 3b). Both the transducer and array latencies were subtracted from the total single-sensor latency to best approximate the actual wave travel time from input to output.

### 2.4. In Vivo Implementation

The in vivo data to demonstrate the methods were collected on one healthy young adult (UW–Madison IRB 2018-0487-CP019). The tensiometer was placed over the right Achilles tendon. The test conditions included both walking (1.33 m/s) and running (2.67 m/s) on a treadmill with the dominant excitation wavelet frequency $f_{H}$ set to 2 kHz. In addition, $f_{H}$ was varied from 1–3 kHz in 500 Hz increments while the participant walked at 1.33 m/s. Data for each condition were acquired for 10 s in duplicate, and the resultant wave velocity profiles were ensemble averaged across multiple (15–26) strides per trial. For each trial, at each normalized percent of the gait cycle, data were eliminated in individual strides which fell outside of a 5% percentile. Additionally, an individual stride would have been excluded if 30% of the total number of data points were outliers, but no strides in these collections met the exclusion criteria.

## 3. Results

### 3.1. Frequency Dependence at a Fixed Speed

**Figure 4:**
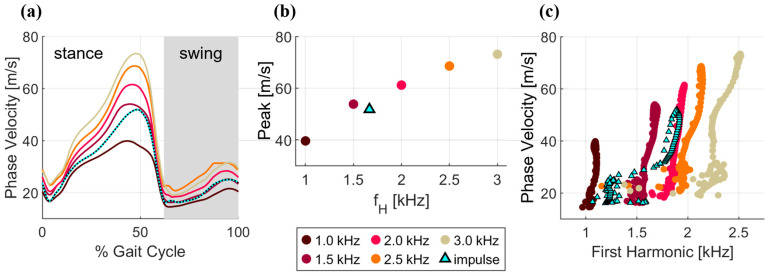
(**a**) Phase velocity as computed with the single-sensor method for walking at 1.33 m/s. The wave speeds computed with impulsive excitation and a dual-sensor approach are overlayed for comparison. The swing phase of gait is highlighted in gray. (**b**) Peak phase velocity vs. the dominant wavelet excitation frequency $f_{H}$. For the impulse, $f_{H}$ was defined assuming the 0.3 ms impulse was half of a sine period, or 1.67 kHz. (**c**) The dominant frequency (first harmonic) of the wavelet excitation response exhibited a slight increase with phase velocity. In contrast, the impulse wave frequencies exhibited nearly a two-fold change over the gait cycle, with the first harmonic ranging from 1 to 2 kHz.

The computed phase velocities were frequency dependent, consistent with the dispersive nature of biological tissues [17]. Most notably, an increase in the excitation frequency resulted in an increase (35 m/s) in the magnitude of the peak phase velocity across the range of excitation frequencies considered (Figure 4a,b). For all frequencies, the standard deviation at peak was no more than 3% of average peak phase velocity across strides.

### 3.2. Comparison of Wavelet and Impulsive Excitation

Wave speeds computed via impulse excitation exhibited a pattern that modulated between phase velocities generated by 1 and 1.5 kHz wavelets. Minimum wave speeds in early stance were ~15 m/s, which is similar to the phase velocity induced via 1 kHz wavelet. The peak wave speed of ~50 m/s during late pushoff was similar to the phase velocity induced via 1.5 kHz wavelet. Likewise, frequency analysis of the first accelerometer signal exhibited a dominant frequency that fluctuated between the frequencies induced by 1–2 kHz wavelets (Figure 4c).

### 3.3. Phase Velocity Changes with Locomotion Speed and Paradigm

**Figure 5:**
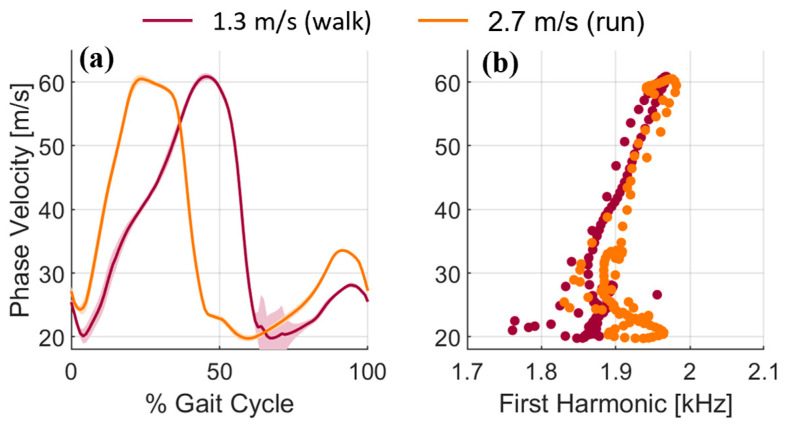
(**a**) Phase velocity during walking (1.3 m/s) and running (2.7 m/s). Shaded areas are ±1 standard deviation about the mean for each percent of the gait cycle. (**b**) First harmonic of the measured wavelet. There were no substantial differences in the first harmonic frequencies between the two locomotion paradigms.

Phase velocity was modulated by load changes in walking and running (Figure 5), similar to prior tensiometry studies [5,11]. The first harmonic frequency was consistent across loading in both walking and running, with no substantial differences between the paradigms.

## 4. Discussion

This study introduced a single sensor tensiometry approach that used shaped wavelet excitations to track in vivo phase velocity in tendons during human locomotion. Phase velocity patterns in the Achilles tendon were generally consistent with wave speed patterns measured via dual-sensor tensiometry. Phase velocity magnitudes did modulate with excitation frequency, reflecting the dispersive nature of tendinous tissue. The single sensor tensiometer has a smaller footprint that is suitable for a broader range of ligaments and tendons, which could enhance the overall utility of tensiometry in practice.

The Achilles tendon exhibited dispersive, or frequency-dependent, behavior, with the phase velocities modulating with excitation frequency over the range (1.0–3.0 kHz) considered (Figure 4b). Such dispersion could arise from the finite thickness and viscoelastic properties of tendons. Analytical models of wave propagation suggest that the finite thickness of a tendon gives rise to wave-guided behavior at lower frequencies [13]. Likewise, the inherent viscosity of soft tissues can result in wave speeds that increase with frequency [17]. The presence of dispersion poses a challenge when impulse responses are used to characterize wave speed in a structure that undergoes variations in stiffness, and hence natural frequency, during movement. Tendon wave speeds computed from impulse responses will thus inherently include variations attributable to both load and frequency, simultaneously. Such an effect is evident in the impulsive wave speed measurements shown here, in which the dominant frequencies increased with loading (from 1.1 to 1.9 kHz). The resulting wave speeds modulated between phase velocities measured with wavelet excitations of 1 and 1.5 kHz (Figure 4a,c). The overall impact of frequency-dependent changes on absolute wave speeds was smaller than the magnitude of load-dependent changes but is nonetheless a factor to consider when selecting an excitation.

While the behavior of wave propagation is understood to be influenced by both the geometry of the structure of interest (i.e., the tendon) as well as the presence of surrounding tissue, literature suggests that for superficial structures, such as the Achilles tendon, surface measurements are dominated by the wave propagating in the stiffest structure [5]. It was observed via ultrasound that across the depth of the skin, subcutaneous fat, and tendon, the structures moved synchronously. This note comes with two caveats. First, this does not disregard that other waves may propagate in surrounding tissue at different speeds; rather that the motion of the tendon causes the surrounding tissue to move with it. Faster or slower waves of smaller amplitude may be present but are small compared to the tendon motion in the observed time window. Second, there is naturally a depth limit to which this technique is applicable; a very deep structure may not sufficiently transmit its motion to the surface for accurate measurement in this way, but for the case presented, surface measurements are appropriate.

The wavelet responses exhibited some load-dependent changes in dominant frequency (Figure 4c), though substantially less than impulsive responses. This is understandable since the wavelet, though centered on a dominant frequency, necessarily contains frequency content outside of this band because the excitation is temporally discrete. For this reason, the dominant frequency measured in the wavelets trended toward the system resonance (for example, the prescribed 3 kHz wavelet had a measured dominant frequency of 2.5 kHz), but variations over the tendon loading cycle were relatively small. It is worth noting that the impulse excitation tended to generate waves with dominant frequencies around the system resonance, as identified in the system characterization (Figure 3a). From this, we can reasonably conclude that tensiometry performed with a shaped excitation about a dominant center frequency may exhibit less variation due to dispersion than a broadband impulse excitation.

An additional advantage when using the single-sensor approach is limiting the distortion of the propagating wave due to the physical measurement contact. Between the transducer input and first measurement location, the wave is free to displace the tendon. Once the wave reaches the first location, the tendon motion is subject to the response of the tendon–sensor system combined, and this behavior is measured at the second measurement location [21]. By limiting the measurement to a single point, the measured response is closer to the unobstructed tendon.

Tensiometry is designed to monitor loading in specific tendons, whereas net ankle torque superimposes contributions from all agonistic and antagonistic muscles active about a joint. Hence, tendon wave speed can modulate across the gait cycle differently than joint torque. This was illustrated by the secondary wave speed peak in late swing, present as expected during walking but also observed here during running, which has been attributed to passive stretch in the triceps surae [5,11].

There are a few limitations to consider with this method. First, the computed phase velocity in the presented results is assumed to be associated with a single frequency. In practice, the shaped waveform was designed to have a dominant frequency, but amplitude modulation naturally includes other frequency content into the signal. Furthermore, the system response introduces distortion and spatial filtering to the signal, such that the measured frequency content is not identical to the excitation. As such, the computed phase velocity will be a close approximation, but not an exact representation, of the speed of a pure sine excitation. Second, the wavelet induces both a forced and free vibrational response in the tendon. The analysis technique presented here is designed to track the forced response by identifying the envelope and phasing of the actuator excitation in the sensor signal. Free response behavior could potentially also be considered by using a sensor in the actuator to characterize the excitation transmitted to the tendon. Third, the single-sensor technique is particularly sensitive to positional accuracy, with only a single dimension used to determine the wave propagation speed. In [20], computation of wave speed using more than two measurement points showed potential improvements in accuracy when compared to the standard dual-sensor technique. While there are advantages to the single-sensor method, the increased importance of accurately constraining spacing is acknowledged. Fourth, this study is focused on a new methodology for measuring phase velocity in biological soft tissues and presents a limited set of experimental results to demonstrate its promise. The in vivo results presented are intended to demonstrate this method, and more extensive human subject testing is needed to provide a more thorough description of the dispersive behavior and repeatability of tendon phase velocity metrics, as well as to draw conclusions about the loading and coordination patterns which drive human movement.

The reduced footprint of a single sensor tensiometer has practical benefits. The more compact design (~30% smaller footprint than a typical tensiometer device) may prove beneficial for applications that have proven challenging to date, such as the shorter tendons of children or other structures such as the quadriceps tendon, hamstring tendons, or myriad tendons and ligaments in the upper extremities. Additionally, since the wave speeds can be computed using only one sensor close to the excitation source, the overall signal-to-noise ratio will be enhanced. Finally, the hardware resources required to acquire the necessary data to perform tensiometry are essentially halved. This further expands on the potential of this implementation to simultaneously quantify the loading patterns on multiple tendons using fewer resources.

## 5. Conclusions

We have demonstrated a single-sensor approach to shear wave tensiometry which can noninvasively track tendon phase velocity under dynamic loading conditions. This approach mitigates dispersion effects, which could prove beneficial when using wave speed to track tendon loading [11]. Further, this technique enables the use of hardware with reduced component requirements and a smaller footprint. By simplifying the sensor, this technique may be more accessible with a lower cost barrier to entry. Additionally, with this approach, the applicability of shear wave tensiometry could be extended to additional structures relevant for biomechanical studies, and in orthopedic and rehabilitation applications.

## Data Availability

Data are contained within the article.
